# Alternative methanogenesis - Methanogenic potential of organosulfur administration

**DOI:** 10.1371/journal.pone.0236578

**Published:** 2020-07-30

**Authors:** Petra Varga, Noémi Vida, Petra Hartmann, Anna Szabó, Árpád Mohácsi, Gábor Szabó, Mihály Boros, Eszter Tuboly

**Affiliations:** 1 Institute of Surgical Research, University of Szeged, Szeged, Hungary; 2 MTA-SZTE Research Group on Photoacoustic Spectroscopy, University of Szeged, Szeged, Hungary; United States Department of Agriculture, Agricultural Research Service, UNITED STATES

## Introduction

Mammalian methanogenesis is principally linked to carbohydrate fermentation by the anaerobic intestinal microflora [1], but alternative pathways for non-bacterial methane (CH_4_) generation have also been described in aerobic living systems [2, 3]. In this respect it has been shown that aerobic CH_4_ can be readily formed from organosulfur compounds at ambient atmospheric pressure and temperature *in vitro* [4]. When using iron (II/III), hydrogen peroxide and ascorbic acid as reagents, the S-methyl groups of methionine and other organosulfur compounds are efficiently converted into CH_4_ [4]. To-date *in vitro* and *in vivo* data have established the possibility of biotic, non-archaeal CH_4_ generation under various hypoxia-linked conditions as well [5–7].

An important novel aspect of this process is CH_4_ bioactivity [6]. While CH_4_ is conventionally believed to be physiologically inert, anti-inflammatory effects were described for exogenous CH_4_ in several experimental hypoxia-reoxygenation conditions [8]. Therefore, these and other data collectively raised the possibility that non-bacterial CH_4_ emissions can be part of an adaptive response to oxido-reductive stress in eukaryotes [6, 7].

Based on this background we hypothesized that the metabolic supply of molecules that contain the sulfhydryl (SH) side chain can influence the biotic methanogenesis. A promising candidate appeared to be allyl isothiocyanate, a naturally occurring biothiol compound, particularly abundant in mustard seed [9]. Biothiols can easily be internalized via dietary intake [10], and theoretically, this route might influence the process of CH_4_ formation. Therefore, the major aim of our study was to investigate the methanogenic potential of organosulfur compounds in model experimental systems of oxido-reductive stress. In this line, the impact of increased biothiol intake (organosulfur-enriched or SH-diet) was examined in association with established markers of redox imbalance in a rodent model of hepatic dysfunction induced by high oral doses of ethanol.

## Materials and methods

### Methane-producing capacity of organosulfur compounds in the hydroxyl radical-generating Udenfriend reaction

These experiments were carried out in 20-ml gastight Supelco vials connected to disposable 20 gauge needles through the septum of the cap. The total volume of the Udenfriend-reaction mixture was 1 ml and it contained the following components: 1 mM H_2_O_2_, 0.5 mM L-ascorbic acid (ASC), 1 mM test compound, 10 μM FeCl_2_ and 6 μM EDTA in 5 mM K-phosphate buffer. Sulfur-containing test compounds included dimethyl-thiourea (DMTU), 2-mercaptoethanol-sulfonic acid (MES), aqueous solutions of white mustard seed (*Brassica hirta*) and coriander seed (*Coriandrum sativum*) extracts and dimethyl-sulfoxide (DMSO) as positive control [4, 5]. All chemicals were purchased from Sigma-Aldrich (Merck, Budapest, Hungary), commercial plant seed products (200–200 mg) from Kotanyi Ltd. were extracted with water (10 mL). The reaction mixture was adjusted to pH 7.4, the tubes were then closed the reaction was initiated by the addition of H_2_O_2_ with a Hamilton syringe through the septum of the cap.

The emanating gas was detected in real-time by photoacoustic spectroscopy (PAS) for 10 min as described previously [11]. Briefly, PAS is a special mode of spectroscopy which measures optical absorption indirectly via the conversion of absorbed light energy into acoustic waves. The amplitude of the generated sound is directly proportional to the concentration of the absorbing gas component. The light source of the system is a near-infrared diode laser that emits around the CH_4_ absorption line at 1650.9 nm with an output power of 15 mW (NTT Electronics, Tokyo, Japan). Cross-sensitivity for common components of breath and ambient air were repeatedly examined, and no measurable instrument response was found for several vol % of CO_2_ or H_2_O vapor. The narrow line width of the diode laser provides high selectivity; the absorbance of CH_4_ is several orders of magnitude greater than that of H_2_O, CO_2_ or CO at 1.65 μm, the wavelength we used. The device was previously calibrated with various gas mixtures prepared by dilution of 100 ppm CH_4_ in synthetic air (Messer, Budapest, Hungary), and it has a dynamic range of 4 orders of magnitude; the minimum detectable concentration of the sensor was found to be 0.25 ppm (3σ), with an integration time of 12 s.

### *In vivo* experiments

The animal experiments were performed on a total of 49 male SKH-1 hairless mice (weighing 30–36 g) in accordance with the National Institutes of Health guidelines on the handling and care of experimental animals and EU Directive 2010/63 for the protection of animals used for scientific purposes. The study protocol was reviewed by the National Scientific Ethical Committee on Animal Experimentation (National Competent Authority of Hungary) and was approved by the Animal Welfare Committee of the University of Szeged (approval No. V/148/2013 and I-74-16/2018).

### Experimental protocols

Whole-body CH_4_ measurements were carried out (see details later) and animals with non-producer CH_4_ status (less than 0.2 ppm CH_4_ changes in comparison to the ambient air) were included. After this pre-screening, the non-CH_4_ producer animals were randomly allocated into 7 experimental groups. Group 1 (n = 7) served as control, these mice were fed with standard laboratory chow (Innovo Kft., Isaszeg, Hungary) for 7 days. In Groups 2 and 3 (n = 7, each) the animals received standard laboratory chow enriched with 10% mustard-seed for 7 days or 14 days, respectively.

Large amounts of oral ethanol induce methanogenesis in non-CH_4_ producer humans and rodents [7], thus in the second part of the *in vivo* study the effect of SH-diet combined with ethanol challenge was monitored. These mice were fed with standard diet for 7 days (Groups 4 and 5; n = 7) or 10% mustard-seed containing chow for 7 or 14 days (Groups 6 and 7; n = 7, each). After this period the animals were provided drinking water (Group 4, n = 7) or water containing 12% ethanol (Groups 5–7) for further 7 days while fed with standard laboratory chow. *Ad libitum* supply of the food and water, or ethanol solution was provided and the daily consumption was recorded.

The whole-body CH_4_ emission of mice was measured using the PAS system in a specially designed closed glass sampling chamber with an internal volume of 180 cm^3^ as reported previously [11]. Prior to the experiments the CH_4_ concentration in the chamber was determined and used as baseline value. The mouse was placed into the chamber which was then sealed and a sample of the chamber gas was analyzed exactly 20 min later. The mouse was then removed, and the measurements were repeated on the subsequent days. The chamber was thoroughly flushed with room air before the next animal was placed in. The whole-body CH_4_ emission was calculated as the difference in the baseline CH_4_ concentration (using 50 measuring point area under the curve) and the cumulative CH_4_ released during the first 5 min of the 20-min sample collection period (using 50 measuring point area under the curve) and then referred to the body weight.

### Determination of the total thiols and non-protein bound thiols in liver samples

After the last gas measurements, the animals were anaesthetized (90 mg/kg ketamine + 25 mg/kg xylazine ip), and tissue biopsies were subsequently taken from the liver. At the end of sample taking, the animals were euthanized by overdosing the anesthetics (300 mg/kg ketamine + 30 mg/kg xylazine ip). After measuring the wet weight, total sulfhydryl groups (TSH) in tissue homogenates (applying Potter homogenizer in PBS-based homogenization buffer containing 0.25 M sucrose, 10 mM Tris, 0.5 mM EDTA at pH 7.4) were spectrophotometrically determined (UV-1601 spectrophotometer, Shimadzu, Kyoto, Japan) by the method of Ellman [12] as well as reduced glutathione content (GSH) in using the method and calculation of Sedlak and Lindsay [13] and the concentrations were referred to tissue wet weight.

### Determination of GSH/GSSG ratio in liver tissue samples

The GSH/GSSG ratio was detected using a Sigma-Aldrich (Merck, Budapest, Hungary, catalogue No. 38185-1KT**)** kit according to the manufacturers instruction at 420 nm using a plate-reader (Fluorostar Omega, BMG Labtech, Ortenberg, Germany). Calculation was made by using the standard calibration curve and values were normalized to wet tissue weight.

### Determination of NADPH oxidase (NOX) activity in liver biopsies

The liver samples were homogenized then 50 μl of re-suspended homogenate was added in Dulbecco’s solution containing lucigenin (10 mM), EGTA (10 mM) and sucrose (900 mM). The NADPH oxidase activity was determined via the NADPH-dependent increase in luminescence elicited by adding 1 mM NADPH (in 20 μl), measured with an FB12 Single Tube Luminometer (Berthold Detection Systems GmbH, Bad Wildbad, Germany). Samples incubated in the presence of nitroblue-tetrazolium served as controls. The protein content of the samples was determined with Lowry’s method. The measurements were performed in triplicates and were normalized for protein content.

### Statistical analysis

Data analysis was performed with the statistical software package GraphPad Prism 5.01 for Windows (GraphPad Software, La Jolla, California, USA). For time-dependent differences within groups, repeated measures Kruskal-Wallis test with Dunn’s multiple comparison test, and for time-dependent differences of multiple groups repeated measures two-way ANOVA on ranks, followed by Bonferroni post-hoc test was applied. Kruskal-Wallis test with Bonferroni post-hoc test was applied to compare multiple groups. In the figures, mean values and standard deviation (SD) are given, p values < 0.05 were considered significant.

## Results

### *In vitro* CH_4_ emission

**Fig 1:**
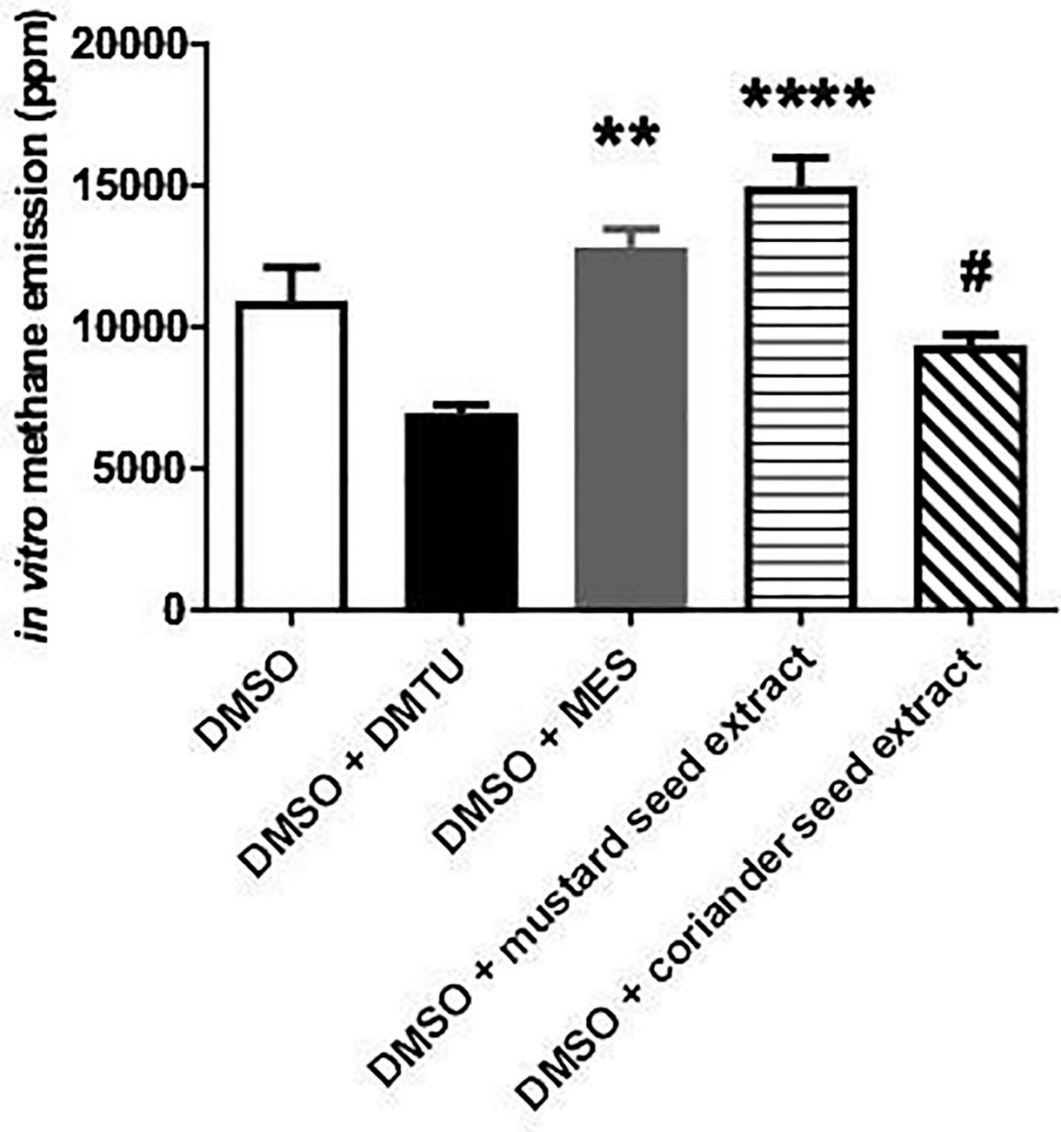
Methane formation in the ROS-generating Udenfriend reaction. Empty bar represents the positive control group, black bar represents the combination of DMSO + DMTU-containing group, grey bar relates to the combination of DMSO + MES-containing group, while white bar filled with light black stripes relates to the combination of DMSO + mustard seed extract-containing group and white bar filled with black diagonal lines to the DMSO + mustard seed extract-containing group. Mean values and standard deviation (SD) are given; **p<0.01 and **** p<0.0001 vs. DMTU+DMSO; #p<0.01 vs. DMSO + mustard seed extract. Statistics: Kruskal-Wallis test with Dunn’s multiple comparison test.

The relative effectiveness of different organosulfur compounds in terms of methanogenesis was detected in a chemical model reaction (Fig 1). DMSO is a potent CH_4_ donor molecule in the presence of reactive oxygen species (ROS) generation [4, 5] Methanogenesis *in vitro* was promptly induced in the DMSO-containing tubes (10925±3139 ppm) while lower CH_4_ emission was detected in the DMSO+DMTU-containing tubes (6954±784 ppm; p = 0.164 vs DMSO). MES, however, augmented the methanogenic capacity of DMSO (12833±1666 ppm; p = >0.9999 vs DMSO). A significant increase was detected after the administration of mustard seed extract (14979±2812 ppm; p = <0.0001 vs DMSO+DMTU), while the released CH_4_ amount was lower in the coriander seed extract-containing tubes as compared to mustard seed extract-induced generation (9364±872 ppm; p = 0.0294). Specifically, the mustard-seed extract kept up the process of methanogenesis during the 10-min observation period.

### CH_4_ release in healthy, unexposed mice

**Fig 2:**
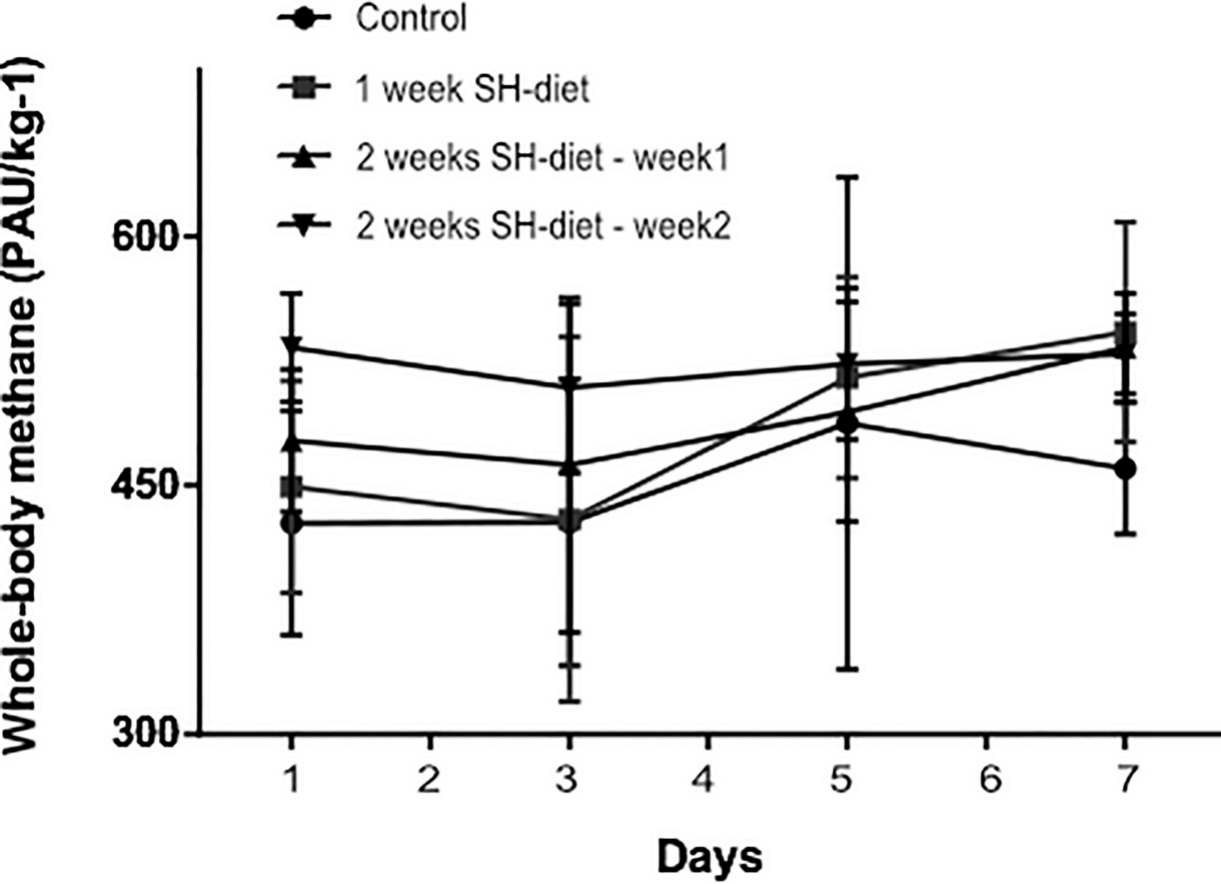
Whole-body CH_4_ profile of the healthy animals during the SH-diet period on days 1, 3, 5, and 7. Black circles with continuous line relate to control group, dark grey square with continuous line to the 1-week SH-diet group. For the sake of simplicity, an individual curve with black, up-pointing triangles with continuous represents for the first half of the 2-weeks SH-diet and another individual curve with black, down-pointing triangles with continuous represents for the second half. Mean values and standard deviation (SD) are given. Statistics: Repeated measures Two-way ANOVA on ranks, Bonferroni post-hoc test.

Based on the *in vitro* findings, we used the mustard seed powder-containing SH-diet for further *in vivo* experiments. The whole-body CH_4_ profile of healthy animals remained steadily low (Fig 2) during the observation period in all experimental groups (Group 1: day 7: 460±39 PAU/kg^-1^ vs day 1: 427±67 PAU/kg^-1^; Group 2: day 7: 454±66 PAU/kg^-1^ vs day 1: 449±63 PAU/kg^-1^; Group 3: day 14: 529±24 PAU/kg^-1^ vs day 1: 476±42 PAU/kg^-1^).

### CH_4_ release in mice exposed to ethanol challenge

**Fig 3:**
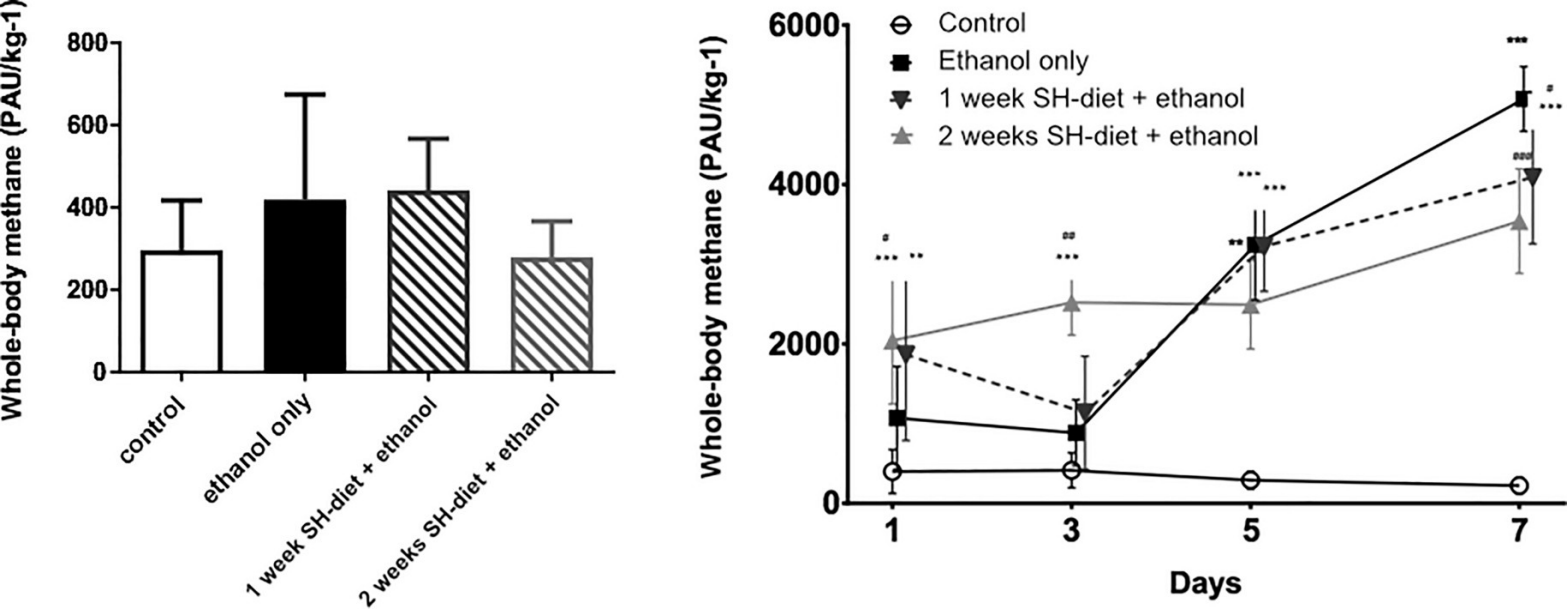
**A**) Baseline whole-body CH_4_ emission in mice on the day before the ethanol-feeding was started. Mean values and standard deviation (SD) are given, significant differences were not observed. Empty bar represents the untreated control group, black bar represents the ethanol-treated group with no antecedent SH-diet, white bar filled with dark grey diagonal lines relates to the 1 week-SH-diet + ethanol treated group, while white bar filled with light grey diagonal lines to the 2 weeks-SH-diet + ethanol treated group. Statistics: Kruskal-Wallis test with Dunn’s multiple comparison test. **B)** Whole body CH_4_ production during the ethanol-feeding protocol on days 1, 3, 5, and 7. Empty circles with continuous line relate to control group, black square with continuous line to the 12% ethanol-treated group with no antecedent SH-diet, dark grey triangles with dashed line to the 1 week-SH-diet + ethanol treated group, while light grey triangles with dashed line to the 2 weeks-SH-diet + ethanol treated group. On the day of measurements all the rats were already given 12% ethanol for 24 hrs, except for those in the control group. Mean values and standard deviation (SD) are given; ***/**/* p < 0.001/0.01/0.05 vs. control; ###/##/# p < 0.001/0.01/0.05 vs. ethanol only. Statistics: Repeated measures Two-way ANOVA on ranks, Bonferroni post-hoc test.

The baseline CH_4_ values did not differ significantly and did not exceed 1547 PAU/kg^-1^ in any of the animals on the night before the ethanol feeding protocol was started (Fig 3A). The dietary ethanol challenge led to a significant CH_4_ release in all groups in comparison to control (Fig 3B). 24h of ethanol consumption was already resulted in slightly elevated CH_4_ output in the ethanol-fed group compared to the control group (day 1: Group 5: 1070±645 PAU/kg^-1^ vs 397±276PAU/kg^-1^) and the whole-body CH_4_ was significantly higher in those groups which were fed with the SH-diet (day 1: Group 6: 1864±1079 PAU/kg^-1^; Group 7: 2042±798PAU/kg^-1^). This pattern was sustained until the 5th day of the ethanol-feeding protocol. Thereafter the highest CH_4_ emanation was detected in animals with no antecedent SH-diet (day 5: Group 5: 3242±693PAU/kg^-1^ vs Group 7: 2492±556 PAU/kg^-1^) and this was sustained thereafter (day 7: Group 5: 5073±406 PAU/kg^-1^; Group 6: 4089±833PAU/kg^-1^; Group 7: 3540±654 PAU/kg^-1^). No changes were observed in the untreated control group during the observation period (day 7: 223±107 PAU/kg^-1^) as compared to those were measured on day 1.

### Total thiols and non-protein bound thiols in liver samples of the healthy, unexposed mice

**Fig 4:**
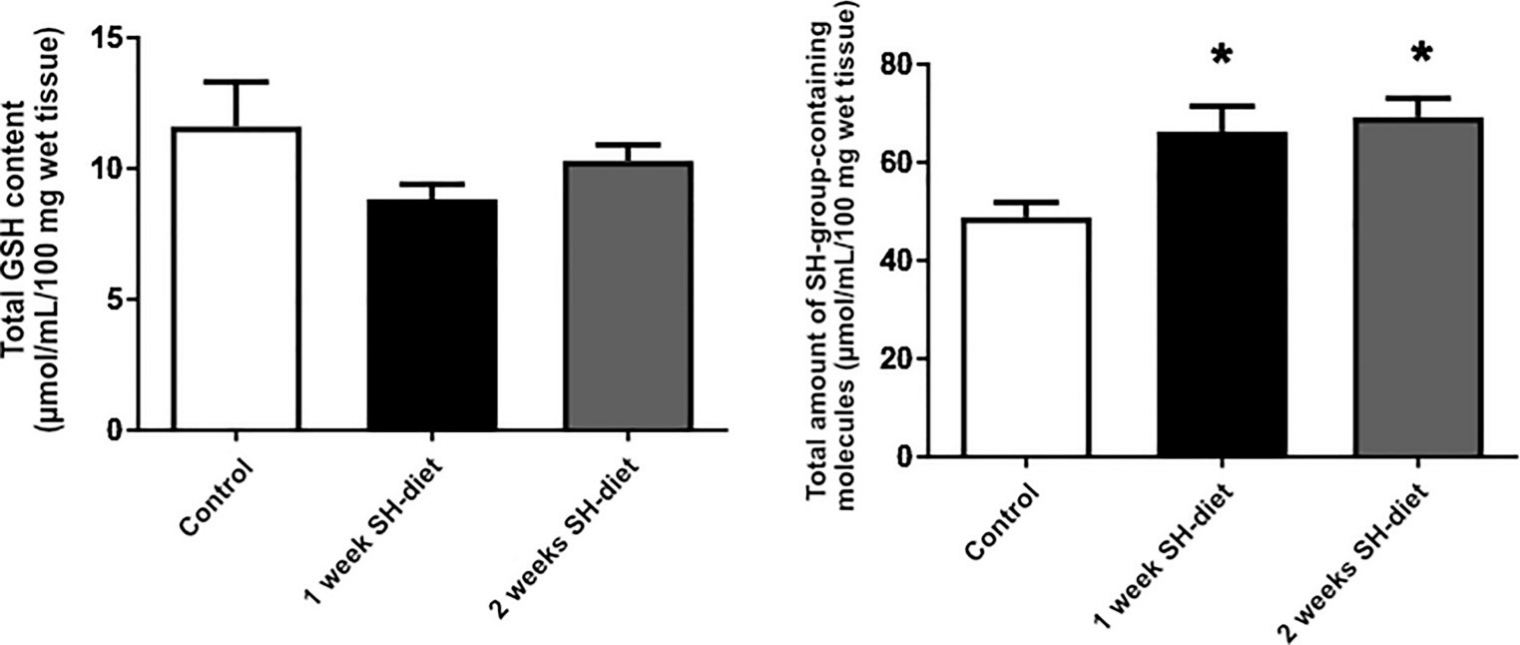
**A)** Total GSH content of liver samples of healthy animals. Empty bar indicates values for the control group, black bar represents data after 1-week SH-diet, while grey bar represents data after 2-weeks SH-diet. Mean values and standard deviation (SD) are given. Statistics: Kruskal-Wallis test with Dunn’s multiple comparison test. **B)** Total amount of SH-group-containing molecules in liver samples of healthy animals. Empty bar indicates values for the control group, black bar represents data after 1-week SH-diet, while grey bar represents data after 2-weeks SH-diet. Mean values and standard deviation (SD) are given, *p<0.05 vs. control. Statistics: Kruskal-Wallis with Dunn’s multiple comparison test.

The liver GSH content did not change in the SH-diet groups as compared to the control group ((Fig 4A); Group 2: 8.83±1.49 μmol/mL/100 mg tissue vs Group 3: 10.3±1.62 μmol/mL/100 mg tissue vs Group 1: 11.61±4.51 μmol/mL/100 mg tissue). Nevertheless, the overall thiol concentration in the liver significantly increased by the SH-diet in both groups ((Fig 4B); Group 2: 66.32±13.47 μmol/mL/100 mg tissue vs Group 3: 69.24±10.07 μmol/mL/100 mg tissue vs. Group 1: 48.86±7.924 μmol/mL/100 mg tissue). In line with these data, the total protein concentration of the samples in Groups 2 and 3 also increased (control vs 2-weeks SH-diet p = 0.0441, *data not shown*) which might also reflect alterations in the SH-containing amino acid metabolism caused by the diet.

### GSH/GSSG ratio in liver samples after ethanol challenge

**Fig 5:**
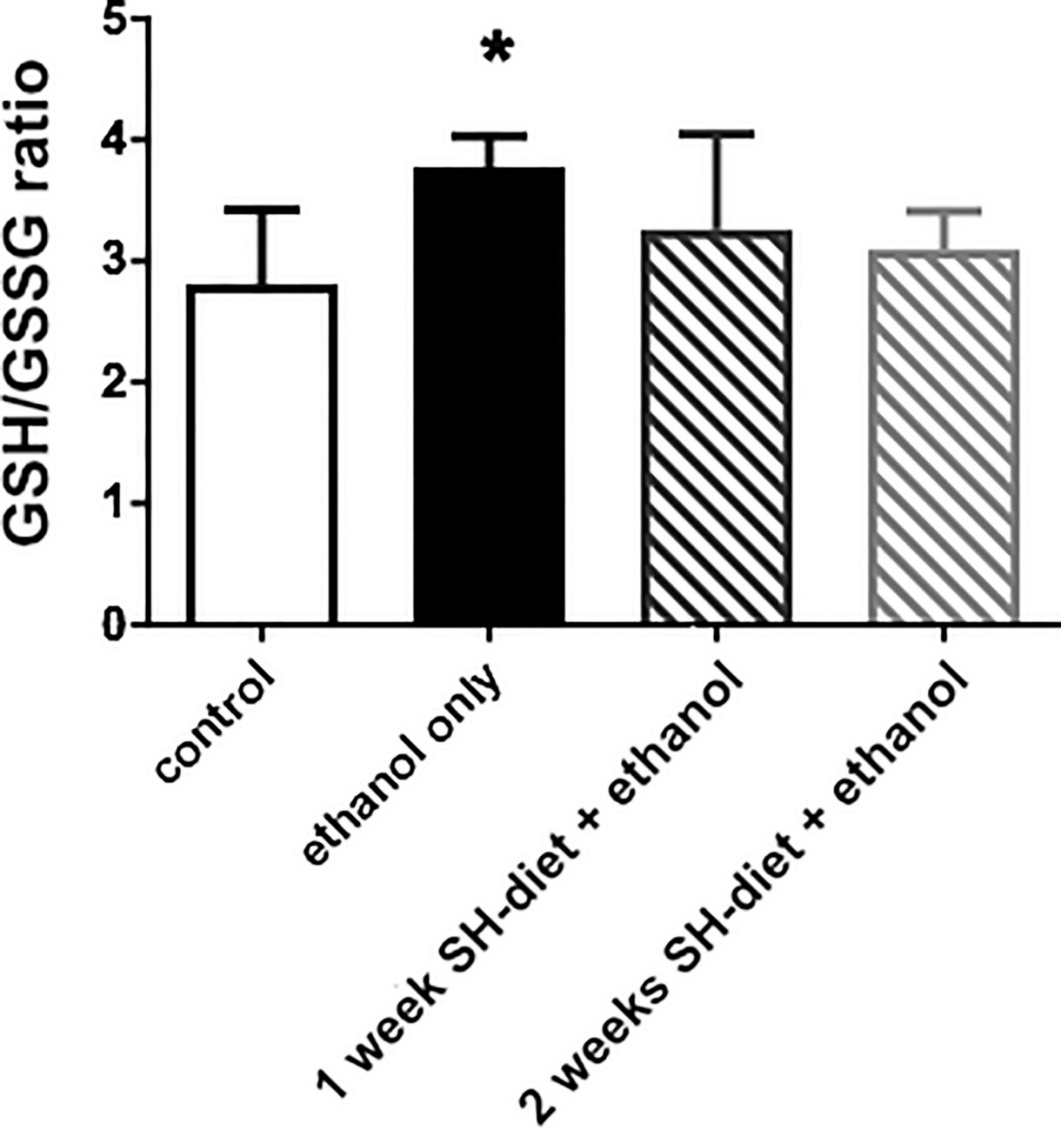
GSH/GSSG ratio in liver samples. Empty bar represents the untreated control group, black bar represents the ethanol-treated group with no antecedent SH-diet, white bar filled with dark grey diagonal lines relates to the 1 week-SH-diet + ethanol-treated group, while white bar filled with light grey diagonal lines to the 2 weeks-SH-diet + ethanol-treated group. Mean values and standard deviation (SD) are given, *p<0.05 vs. control. Statistics: Kruskal-Wallis with Dunn’s multiple comparison test.

The ethanol challenge led to a significant increase in the GSH/GSSGratio as compared to the control group (Group 5: 3.777±0.256 μmol/L/100 mg tissue vs Group 4: 2.809±0.611 μmol/L/100 mg tissue). The 1-week or 2-weeks-SH-diet attenuated this effect significantly and kept the GSH/GSSG ratio at the control level (Group 6: 3.258±0.789 μmol/L/100 mg tissue vs Group 4; Group 7: 3.09±0.317 μmol/L/100 mg tissue vs Group 4) (Fig 5).

### NOX activity of liver samples

**Fig 6:**
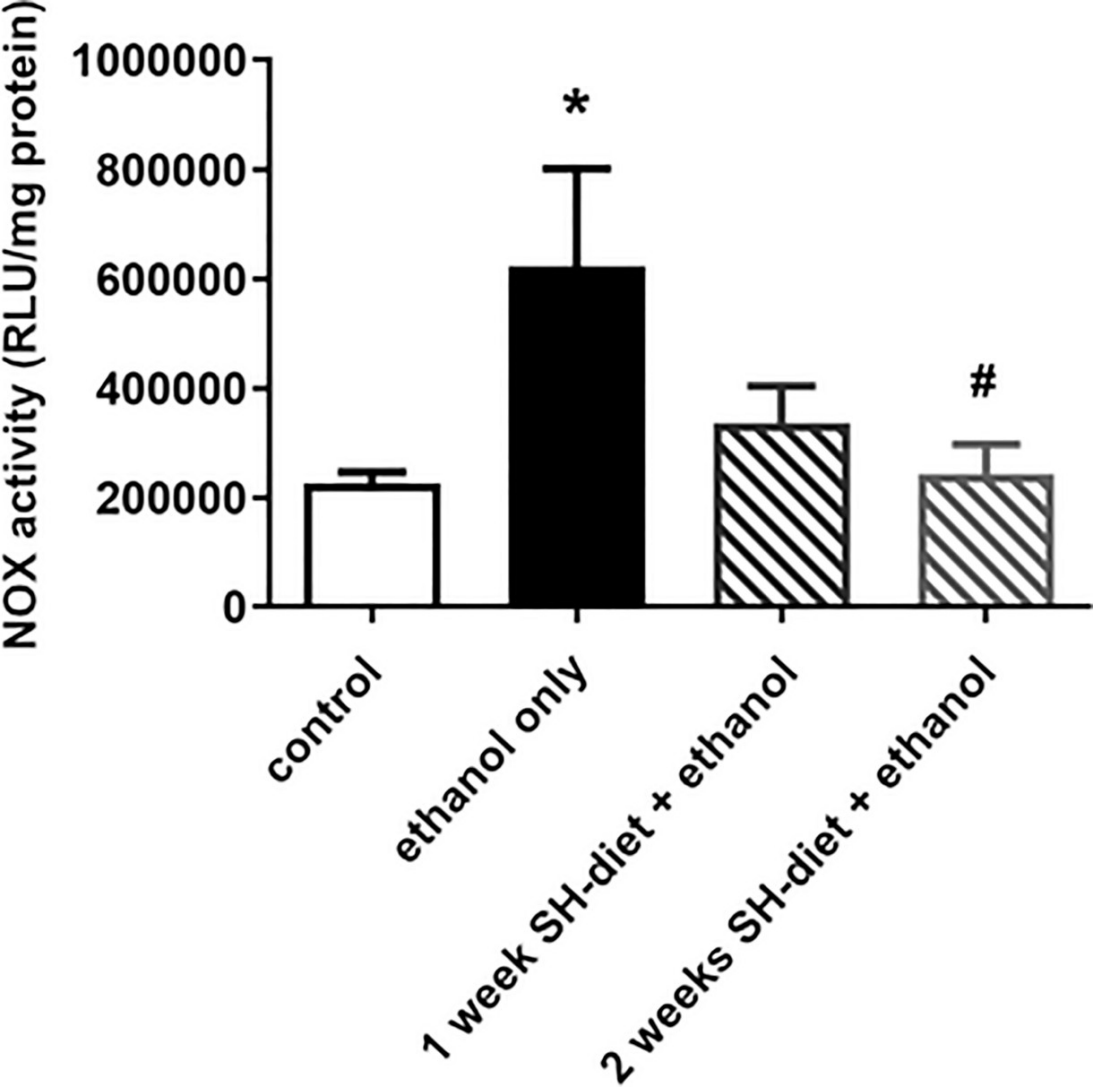
NOX activity in the liver. Empty bar represents the untreated control group, black bar represents the ethanol-treated group with no antecedent SH-diet, white bar filled with dark grey diagonal lines relates to the 1 week-SH-diet + ethanol-treated group, while white bar filled with light grey diagonal lines to the 2 weeks-SH-diet + ethanol-treated group. Mean values and standard deviation (SD) are given. *p<0.05 vs control; #p<0.05 vs. ethanol only. Statistics: Kruskal-Wallis with Dunn’s multiple comparison test.

NOX is a family of membrane-bound oxidoreductase complexes whose main function is the catalysis of the reduction of oxygen (2NADPH +2O_2_—> 2NADP^+^ + 2H^+^ + 2O_2_^-^ —> 2NADP^+^ + H_2_O_2_). Due to ethanol consumption, an elevation in NOX activity was detected as compared to control (Group 5: 622069±474047 μmol/ml/mg protein vs Group 4: 225707±51216 μmol/ml/mg protein, p = 0.0174). This increase was remarkably moderated by the 2-weeks SH-diet as the control level was reached in this group (Group 7: 243191±143215 μmol/ml/mg protein; p = 0.079, p = 0.04 vs Group 5) (Fig 6).

## Discussion

SH-containing amino acids and their metabolites can be involved in the process of *in vitro*, aerobic methanogenesis [4]. Taking these findings into consideration we hypothesized that mustard seed extract could act as a potential source of non-microbial CH_4_ formation. After confirming this possibility, *in vivo* experiments were performed with dietary SH-enrichment in non-CH_4_ producer rodents with or without ethanol stress. Changes in liver thiol concentrations and representative oxido-reductive parameters were detected in order to shed light on the mechanistic aspects of the methanogenic reaction. On the one hand, the concentration of thiol-containing molecules in the liver was significantly increased, suggesting an influence on biothiol tissue storage. On the other hand, the SH-diet *per se* did not influence the net CH_4_ output which might suggested that the onset of oxido-reductive imbalance is needed for an amplified CH_4_ release. As expected, when these animals were involved in the high-dose ethanol-feeding protocol, methanogenesis was induced. The SH-diet remarkably increased the CH_4_ release after 24h of ethanol consumption, and this effect was not observed in animals kept on standard feeding. What is more, the CH_4_ production declined in the SH-enriched diet groups by the end of the ethanol protocol possibly due to pertaining tissue protective events. In contrast, in groups with no extra biothiol feeding, the highest CH_4_ production was reached by the later time point. The time course of the methanogenic reaction was somewhat different to that observed in a previous *in vivo* study [7] but this can be explained by interspecies differences to ethanol-induced adaptation as demonstrated recently by a large-scale work of Adkins *et al*. on cross-species comparisons of ethanol-responsive genes [14].

One of the consequences of alcohol consumption is NADH+H^+^ production which leads to reductive stress and a critical disruption of the cellular redox balance [15]. Althoff and co-workers demonstrated that non-microbial methanogenesis can take place in a biphasic oxidation/sulfoxidation of methyl sulfides, plausible only in highly reductive environment [4]. As sulfoxidation of methyl sulfides is ubiquitous in the environment in the presence of high level of ROS, this chemical route is most likely to facilitate CH_4_ release in living aerobic organisms. This concept can explain the ethanol-induced CH_4_ generation, but further experimental data are clearly needed to shed more light on the underlying organosulfur biochemistry. It should be added that NOX is the major source of ROS and play predominant roles in the pathogenesis of early alcohol-induced hepatic injury [16]. More importantly, the liver NOX activity was significantly elevated after the ethanol challenge; however, it was moderated in the SH-diet groups.

To-date many studies have examined the effects of high intakes of SH amino acids (i.e. methionine and cysteine) on growth and life cycle in rodents [17]. A limited number of studies have also been conducted on the impact on inflammatory responses [18] and the *in vitro* effects of metabolic products of SH-amino acids on human immune cells [19]. Cellular concentrations of biothiols and GSH have been linked to T-cell numbers [20]; the tissue biothiol content responds to SH-amino acid intake by a broad range of intra/intercellular signaling pathways involving metabolization of the essential cofactors leading to GSH production (e.g. vitamin B6, riboflavin, and folic acid). Administration of thiol-containing GSH precursors as n-acetyl cysteine (NAC) or L-2-oxothiazolidine-4-carboxylate (OTC), are of interest in nutrition research [21]. It has been shown that a low-protein diet would suppress GSH synthesis, a situation that is reversed by cysteine or methionine [22]. Despite these supportive data, the impact of a high SH- amino acid diet on established inflammatory responses has not been investigated in any depth in humans or experimental animals.

The anti-inflammatory potential of organosulfur-containing plants such as mustard, horseradish or wasabi is usually linked to the AITC content of these herbs [9, 23] and it has been demonstrated that the nuclear factor erythroid 2–related factor 2 (Nrf2) and NF-κB routes are involved in the signaling pathways [23]. Several lines of evidence indicate that Nrf2 plays a key role in the regulation of cellular SH-amino acid/GSH turnover and controlled GSH/GSSG ratio as well. *In vitro*, Nrf2 regulates the cysteine/glutamate exchange transporter that maintains intracellular GSH levels by allowing cysteine influx [24]. Besides, Nrf2 regulates glutathione peroxidase (GPX2) and glutathione-S-transferase (GST) in human alveolar epithelial cells [25]. In addition, higher levels of GSSG was detected in *Nrf2*−/− alveolar macrophages than in wild-type Nrf-2 cells in response to oxido-reductive stress stimuli [26]. In the current study, we demonstrated a significant contribution of SH-diet on liver biothiol storage as the quantity of molecules with SH residues elevated significantly after 7 days of feeding. Interestingly, the tissue non-protein-bound thiol content (which is represented in 95% by GSH) did not alter in a same manner although the total protein concentration correlated positively with the changes of TSH, indicating that the liver antioxidant capacity was embedded to its proteome, rather than in free GSH. The SH-diet contained a relatively high concentration (10%) of crashed mustard-seed, thoroughly mixed and enriched into the standard laboratory chow which enabled *ad libitum* consumption. Others have already reported on the use of 5 to 10% mustard-seed enriched diet with anti-inflammatory and anti-carcinogenic effects in mice [27]. Upon the emerging reductive stress of the ethanol challenge, these biochemical routes were possibly activated to potentiate the metabolization of GSH from its amino-acid precursors. This possibility is supported by the elevated GSH/GSSG ratio in the liver samples in the conventional diet-fed group after the ethanol protocol, as a higher ratio of GSH to GSSG suggests a reductive environment [28]. This elevation was not present in the mustard-seed diet groups and the GSH/GSSG ratios remained at the control levels. The accumulation of GSH can be linked to ethanol-evoked over-expression of Nrf2 as reported in similar stress conditions [29]. Nevertheless, further studies are necessary to investigate the direct interactions between the inflammatory trigger, non-microbial methane generation, AITC concentration and Nfr2 and GST/GPX2 expression patterns.

## Conclusion

In summary, the present study provides *in vivo* evidence for the possibility of an SH-enriched diet to trigger non-microbial methanogenesis during ethanol feeding. The potential benefit of this approach is to influence a condition associated with redox imbalance, it might be a reasonable choice in cases where tissue hypoxia-related complications are predicted.

## Supporting information

S1 Fig Protocols for the *in vivo* experiments. In Phase I, the effects of SH-diet (laboratory chow enriched with 10% mustard-seed) without ethanol feeding was monitored. The animals in control Group 1 were fed with standard laboratory chow for 7 days. In Groups 2 and 3, the animals received SH-diet for 7 or 14 days, respectively. In Phase II, the effect of SH-diet combined with ethanol challenge was investigated. These mice were fed with standard laboratory chaw for 7 days (Groups 4 and 5) or SH-diet for 7 or 14 days (Groups 6 and 7). After this period, the animals were provided drinking water (Group 4) or water containing 12% ethanol (Groups 5–7) for further 7 days while fed with standard laboratory chow.
(TIF)

S2 Fig Summary of findings. Administration of plant extracts containing organosulfur (SH) moieties can increase the thiol content of the liver without influencing baseline methanogenesis. Ethanol consumption induces hepatic oxido-reductive-stress condition, which is linked to increased methanogenesis, most probably a potentially tissue-protective mechanism. Preceding oral SH feeding can effectively reduce the ethanol-induced hepatic injury.
(TIFF)
