# Supplementary figures and images for: Alternative methanogenesis - Methanogenic potential of organosulfur administration
Source: PLoS One. 2020 Jul 30;15(7):e0236578. doi: 10.1371/journal.pone.0236578 (PMC7392280; doi:10.1371/journal.pone.0236578)

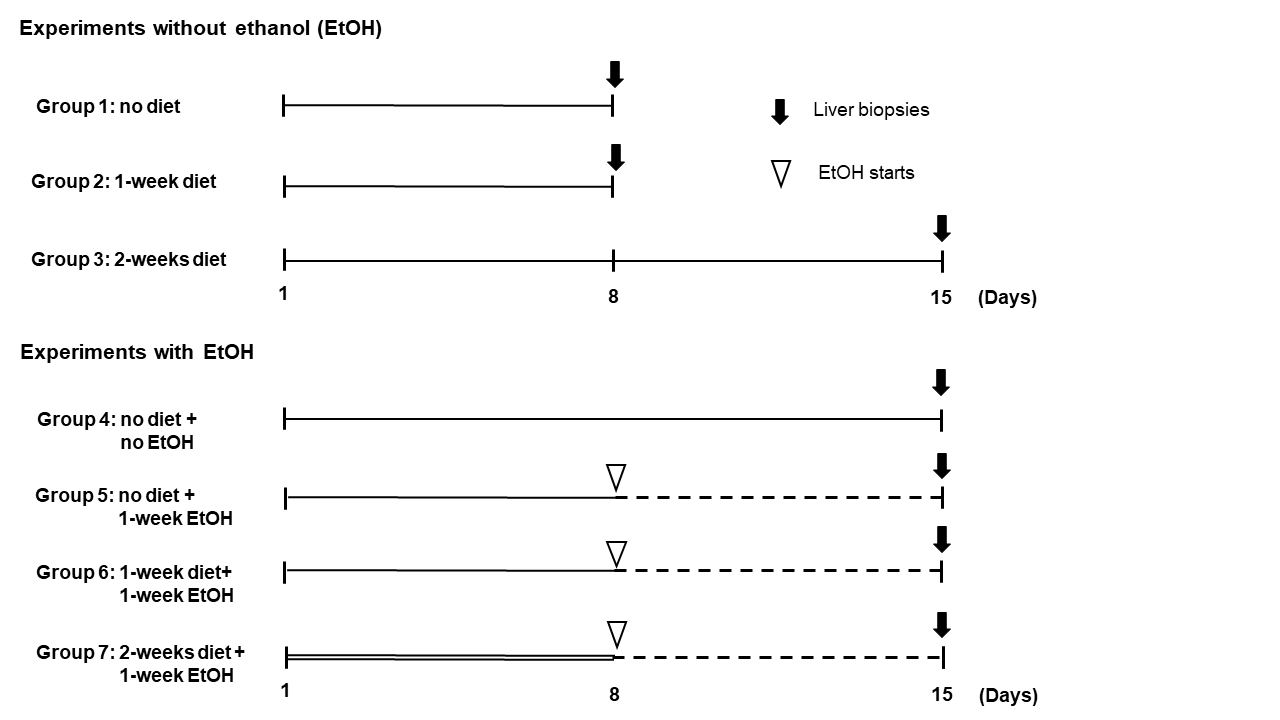

Supplement: S1 Fig — In Phase I, the effects of SH-diet (laboratory chow enriched with 10% mustard-seed) without ethanol feeding was monitored. The animals in control Group 1 were fed with standard laboratory chow for 7 days. In Groups 2 and 3, the animals received SH-diet for 7 or 14 days, respectively. In Phase II, the effect of SH-diet combined with ethanol challenge was investigated. These mice were fed with standard laboratory chaw for 7 days (Groups 4 and 5) or SH-diet for 7 or 14 days (Groups 6 and 7). After this period, the animals were provided drinking water (Group 4) or water containing 12% ethanol (Groups 5–7) for further 7 days while fed with standard laboratory chow. (TIF) [file pone.0236578.s001.tif]

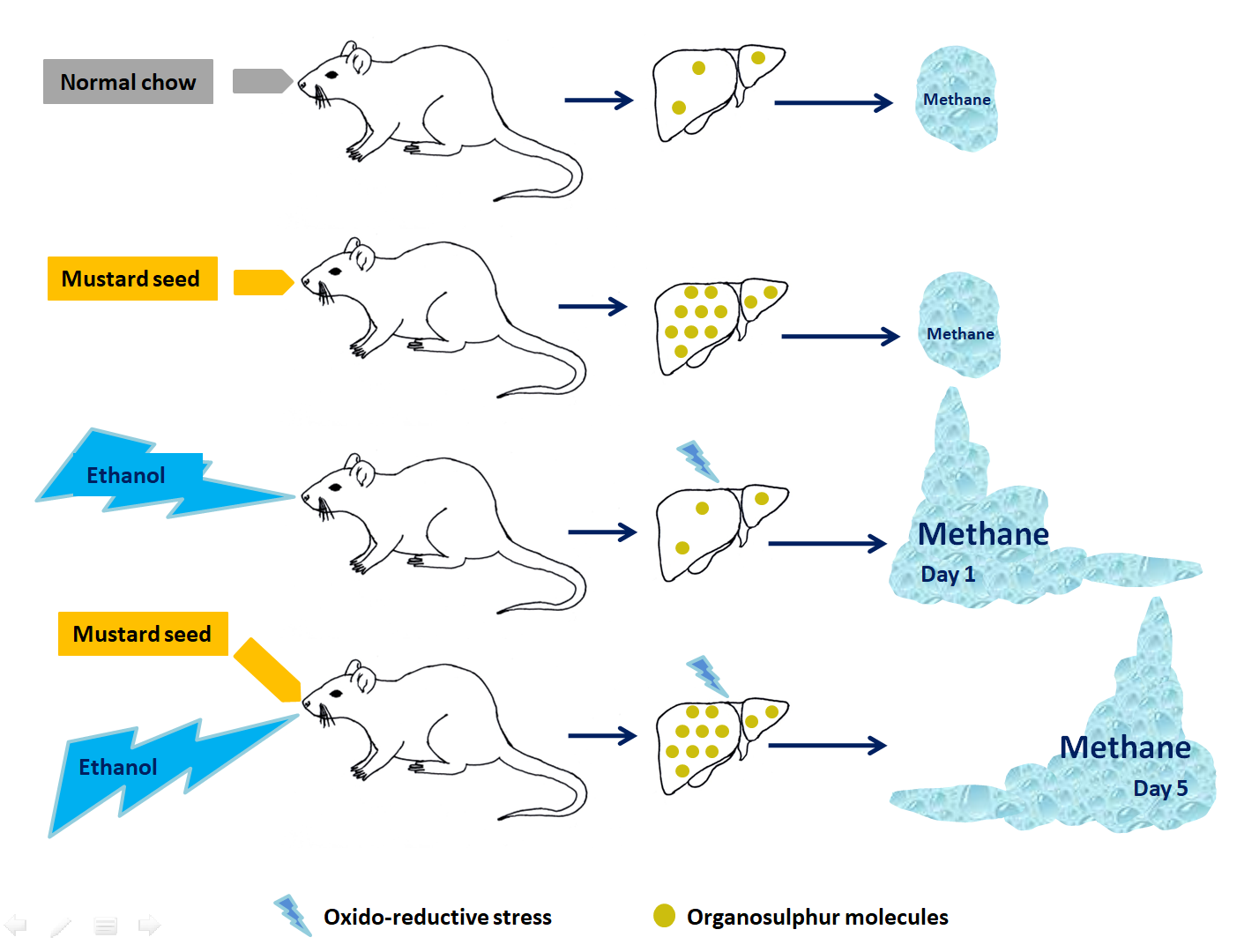

Supplement: S2 Fig — Administration of plant extracts containing organosulfur (SH) moieties can increase the thiol content of the liver without influencing baseline methanogenesis. Ethanol consumption induces hepatic oxido-reductive-stress condition, which is linked to increased methanogenesis, most probably a potentially tissue-protective mechanism. Preceding oral SH feeding can effectively reduce the ethanol-induced hepatic injury. (TIFF) [file pone.0236578.s002.tiff]
